# Development of an Implementation Intervention Using Intervention Mapping to Increase Mammography Among Low Income Women

**DOI:** 10.3389/fpubh.2018.00300

**Published:** 2018-10-26

**Authors:** Linda Highfield, Melissa A. Valerio, Maria E. Fernandez, L. K. Eldridge-Bartholomew

**Affiliations:** ^1^Department of Management, Policy and Community Health Practice, UTHealth School of Public Health, Houston, TX, United States; ^2^Department of Health Promotion and Behavioral Sciences, UTHealth School of Public Health, San Antonio, TX, United States; ^3^Department of Health Promotion and Behavioral Sciences, UTHealth School of Public Health, Houston, TX, United States

**Keywords:** intervention mapping, implementation intervention, consolidated framework for implementation research, mammography, underserved women

## Abstract

**Background:** Although much work has begun to elucidate contextual factors influencing implementation, the specific processes that facilitate and hinder adoption, implementation, and maintenance of evidence-based interventions (EBIs) in clinical settings remains poorly understood. Intervention Mapping (IM) is a systematic process that facilitates planning and design for dissemination, implementation and maintenance of EBIs in practice. IM has been used to guide the design of many health interventions, focusing on program implementation. Less studied is its use to adapt and scale screening interventions within the healthcare clinic setting. This paper describes the development of an implementation intervention using IM to facilitate the adoption, implementation, and maintenance of an EBI designed to increase mammography adherence in healthcare clinics, the adapted Peace of Mind Program (PMP).

**Methods:** IM framework, Step 5, was used to guide the implementation intervention planning. IM guided identification of specific adoption, implementation, and maintenance performance objectives. We formed an implementation intervention planning group consisting of members of the academic team, our community partner and community health workers (CHWs) with substantial experience working on mammography screening programs in federally qualified health centers (FQHCs) and charity clinics.

**Results:** Results are presented by Intervention Mapping task for Step 5 (Program Implementation Plan). We describe how the consolidated framework for implementation research (CFIR) informed the selection of performance objectives, determinants, methods, and practical applications in the final implementation intervention.

**Conclusions:** This paper provides an example of the use of Intervention Mapping Step 5 and CFIR to create an implementation intervention to support EBI scale up of an evidence-based mammography intervention within a specific setting.

**Clinical trials registration number:** NCT02296177

## Background

The research to practice gap is well-documented; only a fraction of evidence-based interventions (EBI) are integrated into practice settings and fewer still are sustained in practice over time (1–5). Although much work has begun to elucidate contextual factors influencing implementation, the specific processes that facilitate and hinder adoption, implementation, and maintenance of EBIs in clinical settings remains poorly understood (6, 7). Further, practitioners' knowledge and expertise is rarely effectively integrated into program design and testing, resulting in programs that may not fit well within the implementation context, or match the needs of the communities they were intended to benefit (4, 8–11). The development of effective implementation strategies should include participatory approaches and be guided by theory. Theory driven D&I interventions that consider individual and systems-level change, can improve the likelihood of adoption, implementation and maintenance of EBIs (12) and support policy and practice changes that improve health outcomes over time. However, few programs to date have used theory to inform their approaches. Davies et al. reviewed 235 D&I studies and found that only 23% used theory to inform the design of their strategies (13). Further, these D&I strategies rarely use multi-level approaches to increase EBI use (14).

There are both few programs available that target mammography adherence in underserved populations specifically and even fewer that use well-defined adoption, implementation and sustainment interventions for mammography EBIs in the U.S. (15). Underserved populations (women who lack insurance or who are underinsured and low-income) have increased risk for late-stage breast cancer diagnosis due to a combination of factors, including lower mammography screening rates overall, high rates of missed screening appointments and lack of timely referral to diagnostic evaluation and treatment in those who screen abnormal (12). Considering the second factor (missing appointments), it has been shown that women who missed screening appointments were more likely to be diagnosed at a later stage of cancer than women who attended their appointments outside of the other two factors (16). This highlights the need for EBIs that improve mammography appointment attendance in underserved women since these women have already addressed the first step of engaging with the healthcare system and scheduling a screening appointment. Gaps in understanding of how best to translate lessons learned from research for integration of EBI's into everyday use–taking into account the local setting and needs of the multiple stakeholders has left many effective mammography programs unused or applied with limited fidelity (17).

Well-designed dissemination and implementation (D&I) strategies are particularly important for the execution of multi-level interventions, which are typically used within complex practice systems such as health care settings to address differences in health outcomes (18). Intervention Mapping (IM) is a systematic process that facilitates planning and design for dissemination, implementation and maintenance of EBIs (19–22) in practice. Intervention mapping has been used to guide the design of many health interventions including a focus on program implementation (12). Less studied is its use to adapt and scale screening interventions within the healthcare clinic setting. This paper describes the development of an implementation intervention using Intervention Mapping to facilitate the adoption, implementation, and maintenance of an EBI designed to increase mammography adherence in healthcare clinics, the adapted Peace of Mind Program (PMP).

## Methods

PMP is a telephone-based EBI to increase mammography appointment adherence (attendance) in underserved women who have scheduled mammography screening appointments. PMP uses a scripted, tailored telephone counseling reminder call which was developed using the Transtheoretical Model of Change to counsel patients through barriers to appointment attendance, such as fear of screening or fear of outcome (4, 12, 23, 24). In addition, PMP engages the patient in active planning for their appointment, such as ensuring the correct paperwork has been completed and that required documents will be brought with the patient (e.g., proof of income) (4, 12, 23, 24). The PMP was designed for use in federally qualified health centers (FQHCs) and charity clinics providing access to mobile mammography services (4, 12, 23, 24). PMP had been previously adapted for underserved women and evaluated using IM [Int Map Adapt and found to effectively reduce appointment no-show rates from 44% (comparison) to 19% in the intervention arm (23, 24). The adjusted odds of a woman in the intervention group attending her appointment were 3.88. The adjusted odds of a woman attending her appointment in the intent-to-treat analysis were 2.31 (23, 24)]. However, previous implementations of PMP lacked a mechanism for taking the program to scale across multiple sites. Our previous studies had focused on development and evaluation of program components, but had not focused on structures necessary to take the program to scale. For this project, our aim was to develop an implementation intervention to support the implementation and scale-up of the EBI in 20 FHQCs and charity clinics in the Greater Houston region, Texas (24). The Intervention Mapping framework, Step 5 was used to guide the implementation intervention planning. Intervention mapping guided identification of specific adoption, implementation, and maintenance performance objectives (who had to do what to implement the intervention). It helps the planning group identify determinants of implementation; *why* clinics (decision makers and staff) or clients would adopt, implement, and maintain the PMP (19).

**Figure 1:**
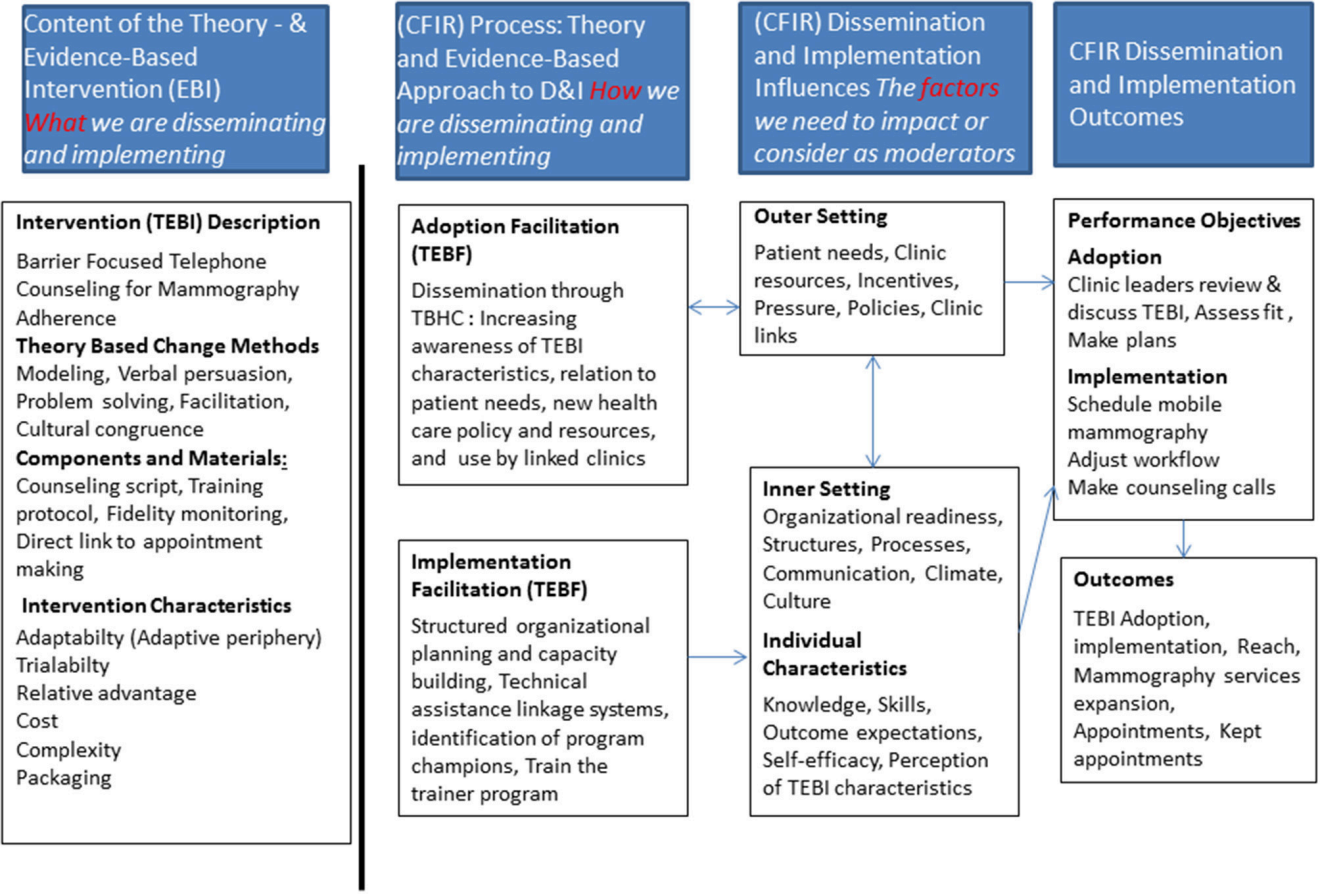
Conceptual framework for development of PMP.

IM allows for integration of theories and frameworks to inform the implementation intervention. In our project we used CFIR to inform the planning process. The CFIR is a meta-framework which includes five domains [intervention characteristics, outer setting, inner setting, characteristics of individuals and process; (6, 25)]. Within these five domains are 39 underlying constructs that may influence implementation and development of clinical guidelines (6). CFIR was used to identify potential contextual factors that may influence the implementation and sustainability of the PMP as shown in Figure 1. Other theories that informed both the selection of determinants of implementations as well as methods for effecting change included Social Cognitive Theory, and Diffusion of Innovation (26, 27).

We formed an implementation intervention planning group to guide the process. The group consisted of members of the academic team, our community partner—the Breast Health Collaborative of Texas leadership and community health workers (CHWs) with substantial experience working on mammography screening programs in FQHCs and charity clinics in the Greater Houston area. Based on previous studies conducted by the team, the experience of planning team members in the community setting, and a review of the literature, we pre-determined that FQHCs and charity clinics were the primary stakeholders for adoption, implementation and maintenance. This project received approval from the Institutional Review Board at the academic institution, protocol number HSC-SPH-14-0269. Per Institutional Review Board review, written informed consent was not required. Women who later participated in the trial and received reminder phone calls gave verbal consent at the outset of the phone call.

## Results of the application of IM step 5 for PMP development

Results will be presented by Intervention Mapping task for Step 5 (Program Implementation Plan). We describe how CFIR informed the selection of performance objectives, determinants, methods, and practical applications included in the final implementation intervention.

### Task 1. identify program adopters, implementers and maintainers

We first identified what stakeholders would be involved in the adoption, implementation and maintenance of the PMP. We then held a brainstorming session with the planning group to answer key questions that would inform the development of the implementation intervention such as: (1) *Who will make the decision to adopt the PMP program in FQHCs or community clinics and who will these decision-makers need to consult?* (2) *Who will implement the program? Will the PMP program require different people to implement different components?* and (3) *Who will ensure that the PMP program is maintained as long as it is needed?* Following the brainstorming sessions, we completed detailed summaries to inform the following tasks and verified our implementers with clinic staff knowledge of FQHC and charity clinic structures. Based on our brainstorming sessions, the planning team determined that the clinic leader would be the adoption decision maker in our participating locations. Clinic leaders typically hold roles such as Executive Director or Chief Executive Officer and are decision makers. In order to adopt the program, the clinic leader would need to meet with the PMP team, review and sign an MOU and assign staff to participate in the program. Adoption performance objectives and determinants are summarized in Table 1.

**Table 1:** Adoption performance objectives and determinants.

**Performance objectives**	**Attitudes about PMP (informed by CFIR domains: intervention characteristics and inner setting)**	**Knowledge (informed by CFIR domain: personal characteristics)**	**Outcome expectations**	**Self-efficacy**	**Normative beliefs (subjective and descriptive)**
Clinic Decision Makers PO1. Agree to participate in the PMP	A.1.a. Perceive that the PMP is easy to adopt and implement A.1.b.Describe PMP as an improvement over what is done now A.1.c. Describe PMP as if the partners (UTSPH and BHC) are here to help A.1.d.Describe PMP as fitting with organizational goals and needs A.1.e. Believe that breast health needs of their patients and community are important. A.1.f. Describe PMP as effective and evidence-based	K. 1.a. Describe the components of the PMP program. K.1.b. Describe the rates of mammography in clinic including no show rates as a problem that needs to be addressed.	OE.1. Expect that the PMP intervention development partners will provide help with program implementation and resources OS.3. Expect this program will provide effective/improved outreach	SE.1.a. Expresses confidence in the ability to do what is expected by the PMP (increase screening capacity, implementation of the PMP, provide a program provide a program champion, assess and expand clinic resources) SE.1.b. Perceive that the clinic is capable of change SE.1.c. Describes the clinic as ready and able for change (perception of organizational readiness)	NB.1. Express belief that other clinics like theirs are agreeing to implement PMP
PO2. Agree to expand mammography services	A.2. Believes that expanding access to mammography service is important for meeting the needs of clients.	K.2.a. Describe the unmet need among clinic patients related to mammography Describe potential availability of staff to expand mammography services available. K.2.b. Describes the steps needed to expand the mammography services.	OE.2. Expect that increased and enhanced mammography services will decrease mortality from breast cancer	SE.2.a. Express confidence in ability to work with partners to increase screening capacity. SE.2.b. Express confidence in ability for clinic to arrange work flow to	NB.2.a. Express belief that other clinics are agreeing to expand mammography services NB.2.b. Express belief that leaders and other decision makers will encourage expansion. NB.2.c.Believes that other centers support new or expanded partnerships with mobile mammography providers NB.2.d.Express belief that providing financial assistance to underserved patients offered through PMP is normative
PO3. Agree to participate in evaluation	Express belief that the evaluation activities are an important element of the PMP program.	K.3.a. Describe the expected outcomes of the program. K.3.b. Describes the Describes the procedures for participating in the evaluation.	OE.3a. Expect that evaluation results will add value to clinic reporting OE.3.b.Expect that evaluation results will add value and status as compared to other clinics OS.3. Believe evaluation results will help clinic support program and garner future funding	SSI.SE.3a. Express confidence in ability to create records needed for evaluation SSI.IS.3b. Believe clinic is a learning environment	NB.3.Express belief that other clinics using PMP will participate in the evaluation.
PO4. Provide a program champion for the PMP	AP.4.Believe that the program champion is an important element of the program.	K.4.a. Explains the role of program champion in PMP.	OE.4. Expect that a program champion will enable the PMP to be implemented and maintained	SSI.SE.4.Express confidence in the ability to recruit a program champion	NB.4. Lists other clinics like theirs that use champions to assist in practice change or program implementation.
PO5. Gain support from stakeholders reaction to the program (care providers, decision makers, navigators/schedulers, patients) (Informed by CFIR Outer setting domains constructs, e.g., patient needs and resources)	A.5.a. Expresses belief that gaining support from stakeholders is an important step in the success of the program. A.5.b. Describes importance of feedback from stakeholders in making revisions and refinements for practice	K.5.za. Describes key points to discuss with stakeholders regarding the PMP program.	OE.5.a. Expect that gaining support from stakeholders such as care providers, patients and managers will ensure the successful adoption and implementation of the program. OE.5.b. Expects that stakeholders who are consulted will develop feelings of acceptance and ownership of the program	SSI.SE.5 Express confidence in their ability to engage stakeholders and engender buy-in	

### Task 2. state outcomes and performance objectives for each stage (adoption, implementation and maintenance)

For this task, the planning group sought to identify who needed to do what in order to adopt/implement/maintain the program. The planning group met and brainstormed answers to key questions such as: “*What do FQHCs and charity clinics have to do in order to adopt PMP?” “What stakeholders does the planning group need to consult in order for PMP to be adopted?” “What levels of approval do the clinics need in order to adopt PMP?”* To better understand and clearly articulate the goals for implementation, we posed the following questions: “*What do the program implementers need to do to implement the essential PMP program components?”* To better understand and articulate maintenance of PMP over time, we needed to more clearly understand what would be required to sustain the program in the clinics. Thus we posed the following questions: “*What do they need to do to maintain the PMP program?”* Our planning team held a brainstorming session and free-listed performance objectives for each. The CFIR domain “process of implementation” was useful in informing potential answers to this inquiry and subsequent selection of implementation performance objectives (what had to be done to implement the intervention).

In the brainstorming session, a facilitator led the team through answering each question and probed the planning group around specific constructs from CFIR and social cognitive theory to make sure the responses were also informed by theoretical and contextual consideration. We determined that the implementation for the EBI would be led by two clinic staff members, the mammography program manager (or clinic staff manager) and a community health worker/patient navigator based on clinic leadership and staff structure related to their overall environment and specifically to their mammography programs. Performance objectives and determinants for implementation are summarized in Table 2.

**Table 2:** Performance objectives and determinants for implementation.

**Performance objectives**	**Attitudes about PMP (informed by CFIR domains: intervention characteristics and inner setting)**	**Knowledge**	**Outcome expectations**	**Self-efficacy/ Skills**	**Social norms**
Patient navigator PO1. Patient navigator agrees to implement the program and attends two-day PMP training.	A1a. Perceives that the PMP is easy to implement A1b. Perceives that PMP scripts are easy to use A.1.c. Perceives that RedCap online system is easy to use and an improvement over current practice. A.1.d. Describes training as though the PMP partners are here to help	K. 1.a. Describes the components of the PMP program. K.1.b. Describes the rates of mammography in clinic including no show rates. K.1.c. Describes requirements of the PMP intervention	OE.1a. Expects that by attending the training he/she will be able to successfully implement PMP OE.b. Expects program champion and clinic leadership will reinforce and acknowledge them for completing the training successfully	SSE.1. Feels confident in ability to attend and learn from training. SSE1a. Expresses confidence to attend PMP training SSEc. Expresses confidence in the ability to do what is expected by the PMP (increase screening capacity, implementation of the PMP, provide a program champion, assess and expand clinic resources)	NB1. Expresses belief that patient navigators at other clinics like theirs are implementing PMP NB1b. Expresses belief that other patient navigators attend training to learn protocols to increase mammography appointment adherence.
PO2. Searches schedule for upcoming appointments	A.2.a. Believes that it is their role to identify upcoming appointments. A.2.b. Describes process of using data systems to identify upcoming appointments as important for PMP implementation.	K2a. Describe steps to searching schedule to identify upcoming appointments. K.2b.Describes the data system of the clinic and PMP program K.2c. Describes protections for patient information	OE.2. Expect that all scheduled women will be identified for receiving PMP.	SSE.2.a. Express confidence in and demonstrates ability to successfully identify all upcoming appointments	NB2a. Express belief that other patient navigators are searching schedules for upcoming appointments.
PO3. Conducts telephone barrier counseling PO3.a. Makes three attempts to reach patient via phone before appointment PO3.b. Asks staging question for PMP PO3.c. Uses active listening protocol when talking with patient PO3.d. Uses barrier scripts to respond to patient concerns	AP.3a.Describe PMP as a protocol-driven intervention AP.3b. Describe PMP as not too complex and fairly easy to implement AP.3c.Describe PMP as better than current practice	K3a. Describe process for conducting counseling. K3b. List staging questions for PMP. K3c. Describe active listening methods.	OE.3. Expect that the PMP will help women keep appointments better than current practice OE.3.a. Expect that mammography can help women detect cancer early when it is more curable OE.3.b. Expect that increasing mammography services and kept appointments will contribute to lowering mortality from breast cancer	SSE.3.Demonstrate skills for initiating conversation SSE.3.a. Demonstrate skills for determining women's intention for keeping appointment SSE.3b. Demonstrate skills for eliciting barriers and using barrier scripts SSE2.c. Demonstrate skills for supporting conversation with active listening SSE.d. Express self-efficacy for conducting telephone barrier counseling and specific skills	NB3a. Express belief that other patient navigators are conducting telephone barrier counseling. NB3b. Express belief that other patient navigators are asking staging questions. NB3c. Express belief that other patient navigators are using active listening with patients. NB3d. Express belief that other patient navigators use barrier scripts to respond to patient concerns.
PO.4. Champions oversee implementation efforts and provide feedback to navigators	A.4. Describes role in overseeing implementation as important and useful for ensuring fidelity	K.4.a. Describes daily and weekly activities associated with Champion Role. K.4.b. Describes steps needed to oversee implementation.	O.E. 4. Expects that through regular oversight and communication, the PMP program will be implemented effectively.	SSE.3. Demonstrates confidence and ability to oversee implementation of PMP.	NB.4. Believes that other individuals with similar positions in other clinics act as navigators to oversee and provide feedback.
PO.5. Champions identify barriers and provide suggestions for overcoming them	A.5. Describes role in identifying barriers as important to the success of the project.	K. 5. Lists potential barriers to implementation and solutions that could address them.	O.E. 5. Expects that the early identification of barriers to implementation will lead to effective solutions that will facilitate continued program use.	SSE.5. Expresses confidence and demonstrates ability to identify problems during implementation and to work with other implementers to resolve them.	NB. 5Believes that other champions like them have a role that includes the identification and resolution of barriers.
PO.6.Champions interact with the research team and clinic leadership as necessary to share and address identified barriers	A.6. Believes that communicating with development and research team is an integral part of their role and important for success.	K.6. Describes protocol for effectively communicating with research team and clinic leadership to address identified barriers.	O.E.6. Believes that if they communicate with the research team and clinic leadership about implementation progress and any barriers, this will lead to effective solutions and program effectiveness.	SSE.6. expresses confidence and demonstrates ability to communicate with leadership.	NB.6. Believes that other Champions also communicate with the research team and with leadership about implementation progress and any barriers to be addressed.

### Task 3. create matrices of change objectives

The next task, development of matrices of change objectives, included the description of very specific objectives for adoption, implementation, and maintenance. First, we identified the determinants for each stage in a brainstorming session where the PMP planning team answered the following questions: “*Why would adopters decide to use PMP?”; “Why would implementers do what is necessary to implement PMP?”*, and “*Why would implementers of PMP do what it takes to make sure the program is continued over time?”* The CFIR also informed the selection of determinants. For example, the CFIR domain, “characteristics of the innovation” (also describe in Diffusion of Innovation) informed the selection of specific attitudinal determinants that were expected to influence both adoption and implementation. These included attitudes about the efficacy, potential fit, and importance of the PMP program. Following the selection of determinants, we created the matrices of change objectives by crossing the identified determinants with performance objectives asking the question: what needs to change in the determinants (e.g., knowledge, skills) for the implementer to accomplish this performance objective. The resulting cells of the matrix represent specific change objectives that form the blueprint of the implementation intervention. The maintenance of the EBI program as practice would require a commitment from the clinic leadership, program manager and community health worker/patient navigator. The performance objectives and determinants for maintenance are summarized in Table 3.

**Table 3:** Performance objectives and determinants for maintenance.

**Performance objectives**	**Knowledge**	**Outcome expectations**	**Skills and Self-efficacy (Personal characteristics)**	**Attitudes about PMP (characteristics of the innovation)**	**Feedback and reinforcement (observability)**
**Maintenance Outcome**: **The clinic decision makers, program champions and patient navigators will maintain delivery of the PMP in their clinic**.					
The program champion will PO1. Discuss with decision makers the continuation of the PMP after funding	K.1.a. Describe processes that will help a program survive in an organization (e.g., inclusion in job descriptions, reward structures, budgets)	OE.1.a. Expect the program to continue to be value added to patients	SSE.1.a. Demonstrate skills for addressing management issues with decision makers SSE.1.a. Expresses self-efficacy for addressing management issues with decision makers	A.1. Describes early successes with the program as evidence of usefulness and reason to continue it. A1.b. Believes that it is important to maintain the program. A.1.c. Expresses continued satisfaction with enhanced services and improved no-show rates	FR.1.b. Ensure access to the RedCap system will be maintained for use by program partners
PO2. Work with decision makers to continue contractual arrangements for increased mammography services.	K.2.a. Describe relevant organizational and inter-organizational processes for writing and administrating contractual agreements	OE.2.a. Expect contractual arrangements are stable and will continue to function as specified OE.2.b. Expect that decision makers will support contractual agreements OE.2.c. Expect that contractual partnerships will contribute to an increase in mammography services	SSE.2.a. Demonstrates administrative skills to follow-up on contracts and work within the clinic administrative structure		FR.2.a. Express satisfaction with contractual partnerships
PO3. Assure that mammography and no show rates continue to be reported (and remain stable or on upward trend).	K.3.a. Describe how to query EMR for program-relevant information K.3.b. Describe how to visualize and share data from K.3.a. to appropriate clinic staff and partners	OE.3. Expect that continued monitoring and evaluation will contribute to likelihood of program continuation	SSE.3.a. Demonstrate administrative skills to monitor data SSE.3.b Express confidence in the ability to monitor, visualize, and present data	A.3.a. Describes how data and feedback on mammography is important to maintain or improved mammography rates and overall clinic performance	FR.3.a. Express satisfaction with enhanced mammography rates. FR.3.b. Express satisfaction with tools and methods for monitoring data
The decision makers (clinic director) will PO4. Approve steps to assure integration of the PMP into normal clinic routines.	K.4.a. Describe the process whereby the program champion will provide decision makers with feedback on PMP integration K.4.b. Describe steps the decision makers plan to take to support the continued use of PMP	OE.4.a. Expects that continued monitoring and evaluation will contribute to likelihood of program continuation OE.b. Expect that integration of PMP into clinic routine practices will lead to a sustained increase in mammography	SSE.4.a. Express confidence in the ability to maintain PMP as a part of clinic routine practices SSE.4.b. Demonstrate the ability to maintain a workforce that is skilled in utilizing PMP practices SSE.4.c. Demonstrate the ability to utilize feedback materials from the program champion to sustain a well-trained staff	A.4. Believe that approving the integration of PMP into normal clinic routines is an important part of their role in improving clinic practices and serving patient needs.	FR.4.a. Expresses satisfaction with enhanced mammography rates. FR.4.b. Express confidence and satisfaction in feedback provided by the program champion FR.4.c. Express satisfaction in how clinic staff utilize PMP practices in clinic routines

### Task 4. design implementation intervention components

The final task for IM for developing implementation interventions includes choosing the change methods and practical applications, designing the scope and sequence for program components and production of materials for influencing program use. The program planning group began this task by considering the determinants and list of change objectives created in Step 3. Next, they reviewed the relevant research and practice literature to confirm, refute, or modify the provisional list of change methods and their practical applications. This task was completed over a period of 2 months where the planning group met in bi-weekly sessions to review the outputs from Step 3, review and discuss the literature and iteratively update the list of change methods and practical applications. To guide our process, we used a combination of the theories diffusion of innovations (28, 29) and social cognitive theory. We were also guided by the constructs of the consolidated framework for implementation research (CFIR) as shown in Figure 1 through the selection of methods for the implementation intervention. We developed a PowerPoint presentation to keep the process organized which was updated at each team planning session and finalized. The presentation contained background information from the needs assessment, original program implementation and evaluation and brainstormed outcomes from each step of the IM process, serving as a complete record of project work which could be easily modified at each session and viewed by team members in remote locations (e.g., phone or internet connection).

The intervention change components (see Table 4), theoretical methods and practical applications for adoption, implementation and maintenance of the PMP program were developed to support the stated change objectives, including presentations, handbooks, training curricula, MOUs and newsletters. Examples of these program materials are available as a supplement to this article. Implementation of the program is supported through the use of a participatory stakeholder group, where clinic program staff participated in regular meetings with the PMP team to review program materials, address any needed adaptations and timeline adjustments, train in the use of the PMP scripts and online system, phase in implementation (clinic staff slowly take over ownership of the reminder phone calls) and ensure active troubleshooting of any program issues during implementation. Implementation is also supported through the use of bilingual community health workers and PMP materials which are available in multiple languages. Through the site visit, PMP staff collect information on language needs of program participants and translate materials accordingly. Implementation is also supported through the use of an online interface programmed in REDCap which guides the community health worker through each patient phone call starting from informed consent and through all intervention components. Using a simple interface, the community health worker is guided through the scripted intervention and advances to the next step by completing either pre-programmed check boxes or open-ended text boxes. The system collects data on informed consent, the patient's stage of readiness to attend their appointment, barriers counseled and logistical planning offered during the phone call.

**Table 4:** Peace of mind program implementation intervention plan.

**Stage**	**Agent**	**Determinants/change objectives**	**Theoretical change methods**	**Practical applications**
Adoption	Clinic Decision Maker	Awareness/Perceptions of PMP Outcome Expectations Skills and Self-efficacy Feedback and reinforcement	PMP program information Persuasion Role Modeling	Email blast to BHC members with PMP informational video and link to pre-adoption survey Webinar to BHC members covering evidence-based approaches to breast cancer prevention, PMP information and adoption steps Adoption meeting held with interested clinics Financial assistance to clinic Assistance with connecting to mobile providers to increase screening (as needed)
Implementation	All	Awareness/Perceptions Outcome Expectations Skills and Self-efficacy Feedback and Reinforcement	Cue to participate Communication Mobilization Organizational Consultation/Planning	Invite clinic staff to participate in stakeholder group (templates for invitation email) Email template for site visit (including requested participants) and site visit questionnaire Site visit planning meeting Program implementation guide, clinic handbook, stakeholder manual and computer assisted PMP scripts reviewed during participatory stakeholder meetings Implementation readiness checklist Stakeholder meetings to support implementation (continue after reminder calls begin). E-newsletter shared with stakeholders
Implementation	Program Champion Navigator	Awareness/Perceptions Outcome Expectations Skills and Self-efficacy Feedback and Reinforcement	Information Persuasion Skill building and guided practice Modeling Monitoring and feedback Technical assistance/capacity building Facilitation Vicarious reinforcement	Face to face training held over two 4 h sessions. Training was submitted to Texas for CEU certification for community health workers and social workers BHC navigators model EBI behavior and provide ongoing implementation support on-site PMP research team available via email, phone and training booster sessions as needed Paperwork processes to provide funds for patients needing financial assistance from PMP
Maintenance	Program Champion Decision Makers	Outcome Expectations Skills and Self-efficacy Feedback and Reinforcement	Information Persuasion Technical assistance	Face to face meeting to discuss maintaining program Program wrap up email with instructions for continued access to program scripts and contact info for technical support Continued access to online PMP scripts Technical support as needed via email or phone

The PMP was implemented over the course of three phases in each clinic. In phase one, the following steps were accomplished: (1) We conducted site assessments with each clinic to understand baseline processes in their mammography programs, (2) Clinic staff were invited to join the participatory stakeholder group, (3) Stakeholder meetings began and reviewed program materials and recommended adaptations to the implementation protocol as needed, (4) PMP training takes place, and (5) Implementation checklist is used to ensure readiness to start PMP phone calls. In phase two, the following steps are accomplished: (1) BHC navigators on-site, provide role modeling of phone calls and support clinic staff as the program begins, (2) Navigators transition reminder phone call scheduling and responsibility to clinic staff over a period of several months and then monitor calls to ensure fidelity, (3) Re-training is provided as needed during this phase and (4) Stakeholder meetings continue with a focus on troubleshooting any implementation barriers and creating e-newsletters to re-inforce program behaviors and highlight program successes. In phase three, clinics take more responsibility for the program and BHC navigators reduce on-site monitoring. Troubleshooting of implementation issues continues. Finally, as clinics move to maintenance, the PMP team holds a meeting with clinic leadership to discuss PMP maintenance plans and provides information on continued access to the PMP online system and technical support. To support maintenance, the PMP online system remains available to all participating clinics.

## Discussion

Poor rates of EBI adoption and low levels of implementation and maintenance, may lead to ineffective or less than expected impact on health poor outcomes when translating EBIs to practice in the community (19). The research to practice gap will persist until successful models are developed to support adoption, implementation and maintenance of EBIs within real-world settings. Practitioners and investigators have called for better descriptions of the development of implementation interventions to facilitate replication and refinement of EBI implementations and dissemination (30–32). However, there are few published studies which provide information on the process used to develop implementation interventions or how implementation science frameworks, such as CFIR, can inform implementation intervention planning (14, 31, 33). Neta et al. (22) note that despite many calls for research showing the process or frameworks used to develop implementation interventions, it is typically not at all apparent how planners took these issues under consideration while planning their programs (e.g., the “what” and “how”) (22). The authors further note that systematic approaches, including IM, could address this need, especially when used in conjunction with theory (22). This paper provides an example of the use of Intervention Mapping Step 5 and CFIR to create an implementation intervention to support EBI scale up of an evidence-based mammography intervention within a specific setting (FQHCs and charity clinics). A recent systematic review of studies using CFIR found only two that had fully used the CFIR in the pre-implementation phase (34). We found that the inclusion of CFIR determinants in the planning process can facilitate critical implementation intervention design and development, increasing the likelihood of successful dissemination and implementation (34). The development of the implementation intervention resulted in the identification of key determinants that we then created specific strategies and methods for addressing through training and targeted messaging for adopters, implementers and for promotion of program maintenance. We further hypothesized that the use of a participatory stakeholder group would support implementation based both on our conceptual framework and from discussions in the brainstorming sessions about clinics' need for implementation support and to help ensure fidelity of implementation. One of the limitations to our participatory approach was balancing the amount of time required from our community partners to participate in brainstorming and planning sessions. Our team addressed this challenge by focusing our time with community partners on brainstorming activities. We spent additional time outside these meeting sessions working on transcription and translation of the brainstorming materials into EBI components which were then reviewed by our community members. An additional challenge was in getting all levels of FQHC and clinic staff to participate in these planning sessions. Clinic staff have many responsibilities and are not always able to take time away from the office for planning meetings, especially unpaid. We addressed this by working with a community partner who was knowledge of our local clinics and with community health workers who had previously worked in a number of the local clinics. In an ideal setting, we would have had clinic leadership directly participate in the planning sessions.

The Peace of Mind Program developed in this project was adapted from an existing EBI and previously tailored to our local community context for specific mammography barriers. In evaluating the EBI effectiveness, the implementation of the program had been highly tailored to that environment. Further, based on our knowledge of FQHCs and charity clinics in the Greater Houston area, we knew there was heterogeneity in the clinic environments, staffing and mammography program processes. Therefore, we hypothesized that adding a structured, theory-based implementation intervention more broadly relevant to FQHCs and charity clinics to the EBI would be necessary for successful scale-up within this specific context. A recent systematic review of the research-practice gap in primary care settings supports this hypothesis. Lau et al. (35) found overlap with EBI adoption, implementation and maintenance and CFIR constructs used in this project to develop the implementation intervention (what Lau et al. refer to as contextual factors). Additionally, the review highlighted that these “contextual factors” are often notably absent from research and frequently fail to be acknowledged, described or taken into account during implementation or program planning (35). The PMP is currently being evaluated using a non-randomized controlled stepped wedge trial in 16 FQHCs and charity clinics in the Greater Houston area. IM Step 6 was used to guide the development of the evaluation plan for PMP, including measures specific to adoption and implementation within clinics participating in the trial. A full description of the development of the evaluation plan for PMP is beyond the scope of this manuscript. For further information on the evaluation of PMP, we refer readers to Highfield et al. (24), which details the protocol for the PMP trial (24). Briefly, reach of PMP is being measured using Google Analytics tracking from BHC outreach events described above (e.g., email communications, webinars) and through the collection of participation logs for events. Adoption and implementation are being measured through the use of a validated survey of CFIR constructs which was adapted for this project. Implementation is also being measured through our REDCap interface, which tracks navigator's use of the EBI staging question. Evaluation results from the trial are expected in late 2018 and will be able to provide further insight into the effect of our implementation intervention on program implementation, maintenance, fidelity and outcomes (appointment attendance). While full evaluation of the program is underway and will be reported elsewhere, a total of 16 clinics with 24 operating sites, providing mammography services to over 4,500 women during our project period adopted PMP. These clinics served a diverse population of Caucasian, Hispanic, African American and Vietnamese women (all underserved). We anticipate that the program will lead to an increase in mammography screening in participating clinics as a result of the EBI components focused on assessing current screening goals, relationships with mobile providers and serving as a bridge between clinics and providers. Increases in screening in underserved women are important as screening serves as the first step in the pathway to breast cancer disparities (16).

In addition, we are monitoring appointment adherence (no-show rates) along with appointment cancellations, re-schedules, patients turned away for incomplete paperwork and other reasons why a woman may not ultimately complete her mammogram appointment. We believe that assessing the EBI against these additional factors will provide a more complete picture of screening outcomes and barriers. To our knowledge, this is the only paper to date which has applied Intervention Mapping in conjunction with the constructs of the CFIR framework and behavioral theories to develop a systematic implementation intervention for the scale up of a mammography EBI in FQHCs and charity clinics. While this paper is focused specifically on mammography screening, the approach we designed for implementation and the protocols and program materials could serve as a guide for others interested in developing similar programs.

## Conclusions

EBIs which are tested and available for scale up may benefit from use of a structured implementation intervention process. In addition, this paper may provide useful insights for others interested in bridging CFIR, health behavioral theories and scaling up EBIs in community settings, particularly those related to mammography screening in healthcare settings and will facilitate use of the IM steps to support the systematic review and addressing of context specific needs for adoption, implementation and maintenance of EBIs into practice.

## Ethics statement

This study was carried out in accordance with the recommendations of UTHealth Committee for the Protection of Human Subjects. The protocol was approved by the UTHealth Committee for the Protection of Human Subjects. All subjects gave verbal informed consent in accordance with the Declaration of Helsinki.

## Author contributions

LH conceptualized the study, carried out the study and lead the writing of the article. LE-B conceptualized and served as the mentor for the study. MV and MF assisted with study development and manuscript writing.

### Conflict of interest statement

The authors declare that the research was conducted in the absence of any commercial or financial relationships that could be construed as a potential conflict of interest.
